# GSDMD‐Deficient G‐MDSCs Exert Profoundly Suppressive Activity to Relieve MPTP‐Induced Parkinson's Disease

**DOI:** 10.1111/cns.70626

**Published:** 2025-10-13

**Authors:** Qi Wu, Fangzhou Liu, Min Gu, Junjie Wei, Guotao Lu, Li Qian, Xiaobo Li, Weijuan Gong

**Affiliations:** ^1^ Department of Basic Medicine, School of Medicine Yangzhou University Yangzhou China; ^2^ Department of Gastroenterology, Affiliated Hospital Yangzhou University, Yangzhou University Yangzhou China; ^3^ Department of Neurology, Affiliated Subei People's Hospital Yangzhou University Yangzhou China; ^4^ Univeristy Key Laboratory of Jiangsu Province for Nucleic Acid & Cell Fate Regulation (Yangzhou University) Yangzhou China

**Keywords:** ACT001, G‐MDSCs, GSDMD, MPTP, Parkinson's disease

## Abstract

**Aims:**

Myeloid‐derived suppressor cells (MDSCs) are elevated in Parkinson's disease (PD), but their functional role remains unclear. This study investigated whether GSDMD deficiency enhances the immunosuppressive activity of granulocytic MDSCs (G‐MDSCs) to mitigate PD progression.

**Methods:**

Flow cytometry and Western blot analyzed G/M‐MDSCs in 37 PD patients and 21 controls. An MPTP‐induced PD mouse model was used to assess GSDMD‐deficient G‐MDSCs through behavioral tests, immunohistochemistry, and adoptive transfer experiments. The NLRP3 inhibitor ACT001 was evaluated for therapeutic potential.

**Results:**

PD patients and MPTP‐treated mice showed increased peripheral G‐MDSCs with reduced NLRP3/GSDMD activation. GSDMD knockout mice exhibited attenuated PD symptoms, reversed by MDSCs depletion. Adoptive transfer of GSDMD‐deficient G‐MDSCs suppressed microglial activation and improved motor function. ACT001 enhanced G‐MDSCs immunosuppression and alleviated PD pathology.

**Conclusion:**

GSDMD deficiency promotes immunosuppressive G‐MDSCs that inhibit neuroinflammation and PD progression. Targeting GSDMD to modulate MDSCs' function represents a novel therapeutic strategy for PD.

## Graphical abstract

This study demonstrates that GSDMD deficiency leads to the induction of G‐MDSCs, which exhibit immunosuppressive activity that alleviates the progression of Parkinson's disease (PD). Treatment with ACT001 in PD mice resulted in an increase in G‐MDSCs in the brain. GSDMD‐deficient G‐MDSCs or inhibition of GSDMD activation in MDSCs may offer promising therapeutic strategies for PD.

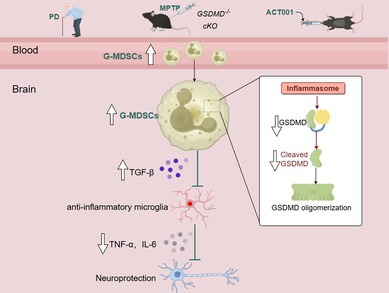

## 1 Introduction

Parkinson's disease (PD) is a progressive neurodegenerative disorder characterized by motor symptoms, including bradykinesia, rigidity, and tremor, and other non‐motor disturbances. Typical changes in pathology include the loss of dopaminergic neurons in the substantia nigra (SN) and decreased dopamine concentrations in the striatum, with the presence of α‐synuclein protein inclusions (Lewy bodies or Lewy neurites) [1, 2]. So far, abnormal immune functions and inflammation have been identified in patients with PD, as demonstrated by dysbiosis and gut inflammation [3]; increased pro‐inflammatory T cells, macrophages, and cytokines in peripheral blood; increased blood–brain barrier (BBB) permeability, with enhanced peripheral cell infiltration of the central nervous system (CNS); and chronic neuroinflammation with activated microglia and pro‐inflammatory cytokines [4, 5]. Obtaining new agents to regulate immune function can suppress inflammation to inhibit the progression of PD.

Myeloid‐derived suppressor cells (MDSCs) are a heterogenic population of immature myeloid cells with immunosuppressive effects. They undergo massive expansion during chronic inflammation and tumor progression. Under conditions of sustained growth factor and inflammatory mediators, neutrophils and monocytes enter an immature phenotype; that is, granulocytic MDSCs (G‐MDSCs) and monocytic MDSCs (M‐MDSCs). Both MDSCs have weak phagocytic activity: increased expression levels of death receptor 5 (DR5) and programmed cell death‐ligand 1 (PD‐L1); enhanced reactive oxygen species (ROS) and nitric oxide production; and high expression levels of arginase (ARG1), prostaglandin E2, and anti‐inflammatory cytokines (IL‐10 and TGF‐β1). MDSCs can suppress the bioactivity of effector T cells and pro‐inflammatory macrophages and increase regulatory T cell function [6, 7]. Increased MDSCs in peripheral blood were identified in patients with PD [8, 9], but the role and significance of these MDSCs in PD progression are still unclear.

The activation of GSDMD is an end‐stage event of inflammasome activation, and it results in cell pyroptosis and the release of IL‐1β and IL‐18 [10, 11]. Given that GSDMD is widely present in immune and parenchymal cells, activating it inevitably exacerbates local inflammation. However, abnormal expression or activation of GSDMD in immunosuppressive cells, such as MDSCs or Treg cells, has not been clarified. The authors previously observed a substantial increase in G‐MDSCs in GSDMD^−/−^ mice. In the present study, whether these GSDMD‐deficient G‐MDSCs could limit PD progression via exerting their immunosuppressive activity was explored.

## 2 Materials and Methods

### 2.1 Patients

Thirty‐seven patients with PD were recruited to collect peripheral blood. These patients were diagnosed in accordance with the Movement Disorder Society Clinical Diagnostic Criteria for PD (2015). All samples were obtained on the basis of the ethical requirements of Subei People's Hospital, Jiangsu Province (No. 2024ky080). Informed consent was obtained from all participants. The clinical characteristics of patients with PD are listed in Table S1.

### 2.2 Mice and PD Model

GSDMD‐knockout (KO) mice (*GSDMD* ^−/−^), *GSDMD* ^flox/flox^ mice, and *S100A8* ^cre^ mice of C57BL/6 background were established by Gempharmatech (Nanjing, China). The *GSDMD* ^flox/flox^ mice were crossed with S100A8^cre^ mice to generate conditional KO mice (cKO) with GSDMD‐specific deficiency in myeloid cells. Animal experiments were conducted in accordance with the Guidelines for the Care and Use of Laboratory Animals in China and approved by the Ethics Committee of Medical College of Yangzhou University (YXYLL‐2020‐67).

A subacute PD mouse model was established using MPTP. 12‐week‐old male and female mice (25 ± 2 g, equal numbers of each sex) received intraperitoneal injections of MPTP (30 mg/kg per dose) every 24 h for 7 days. All injections were performed in a well‐ventilated cabinet to ensure safety and minimize exposure.

### 2.3 Reagents and Antibodies

Antibodies against α‐synuclein (D37A6), GSDMD (E9S1X), NLRP3 (D4D8T), caspase‐1 (E9R2D), cleaved GSDMD (Asp276) (E3E3P), IL‐1β (3A6)/(D3U3E) and Arginase‐1 (D4E3M) were purchased from Cell Signaling Technology (Boston, MA, USA). Antibodies against TH (GB11181‐100), TNF‐α (GB11188‐100) and IL‐6 (GB11117) were procured from Servicebio (Wuhan, China). The Iba1 (ab178846) antibody was obtained from Abcam (Cambridge, UK). Anti‐mouse DR5 (CD262) was acquired from BioXCell (New Hampshire, USA). Antibodies against mouse CD11b (M1/70), Gr‐1 (RB6‐8C5), Ly6G (S19018G), Ly6C (HK1.4), PD‐L1 (10F.9G2), CD45 (30‐F11), TGF‐β1 (TW7‐16B4), IL‐10R (1B1.3a) and human CD33 (5D3), HLA‐DR (L243) and CD11b (ICRF44) were purchased from BioLegend (San Diego, CA, USA). 1‐Methyl‐4‐phenyl‐1,2,3,6‐tetrahydropyridine (MPTP, HY‐15608) and ACT001 (HY‐128861A) were obtained from MedChemExpress (New Jersey, USA). LPS was acquired from Sigma–Aldrich (Darmstadt, Germany).

### 2.4 Behavioral Experiments

#### 2.4.1 Rotarod Test

The motor coordination of mice was evaluated using a rotating device. Prior to drug administration, all mice were trained on the rotarod apparatus from 5 to 30 rpm within 300 s. The training process was conducted more than three times to keep the mice on the apparatus. After MPTP treatment, the tests were performed as before, and fall latency was recorded.

#### 2.4.2 Open‐Field Test

A black plastic box (40 cm × 40 cm × 40 cm) was used for the open‐field test. Before the test, all mice were adapted to the box for 30 min. Their movements were recorded for 5 min. The traveled distance, resting time, and fine motor activities (e.g., sniffing) were recorded.

### 2.5 Immunohistochemistry

After sections underwent antigen retrieval, they were treated with hydrogen peroxide to block endogenous peroxidase activity. They were then incubated with non‐specific staining blockers, and primary and secondary antibodies were successively added. The sections were developed using DAB solution (AR1027‐3, BOSTER) and further stained with hematoxylin. All histochemical results were photographed using an Eclipse 80i microscope (Nikon, Tokyo, Japan).

### 2.6 Immunofluorescence

Tissues were embedded in OCT and cut into sections of 25 μm thickness. The sections were sealed with 5% BSA and 0.3% Triton X‐100 and incubated with primary and secondary antibodies sequentially. Then, they were counterstained with DAPI. The fluorescence results were captured using an AxioScope 5 (Zeiss, Jena, Germany).

### 2.7 Western Blot

Tissues or cells were lysed and centrifuged to collect the suspension. The proteins of the suspension were separated by SDS‐PAGE and transferred to a PVDF membrane. After membrane blocking, the primary and secondary antibodies were incubated in order. Immunoreactive bands were detected by routine chemiluminescence.

### 2.8 Isolation of Single Cells

Tissues were isolated and dissected aseptically, cut into small pieces, and digested with 0.125% trypsin at 37°C. After centrifugation, single cells were plated into T75 flasks and cultured in DMEM/F‐12 medium containing 1% penicillin/streptomycin and 10% FBS at 37°C in a 5% CO_2_ incubator for further analysis.

### 2.9 Flow Cytometry

Cells were labeled with fluorescence‐conjugated antibody and analyzed using a BD FACSVerse (BD Biosciences, New Jersey, USA). For the detection of intracellular cytokines, the cells were incubated with a Golgi blocker and stained with surface antibody, followed by fixation, permeabilization, and incubation with intracellular detection antibody.

### 2.10 Statistics

All data were analyzed using GraphPad Prism software. All data followed a normal variable distribution and were checked by the Shapiro–Wilk test. The bar was represented as the mean ± SEM. Unpaired student's *t*‐test was used to compare two groups. One‐way ANOVA with Tukey's post hoc tests was used to compare more than two groups. All experiments were carried out at least three times. Significance is indicated by *p < 0.05, **p < 0.01, ***p < 0.001 and ****p < 0.0001.

## 3 Results

### 3.1 Increased G‐MDSCs in the Periphery of Individuals With PD

**FIGURE 1:**
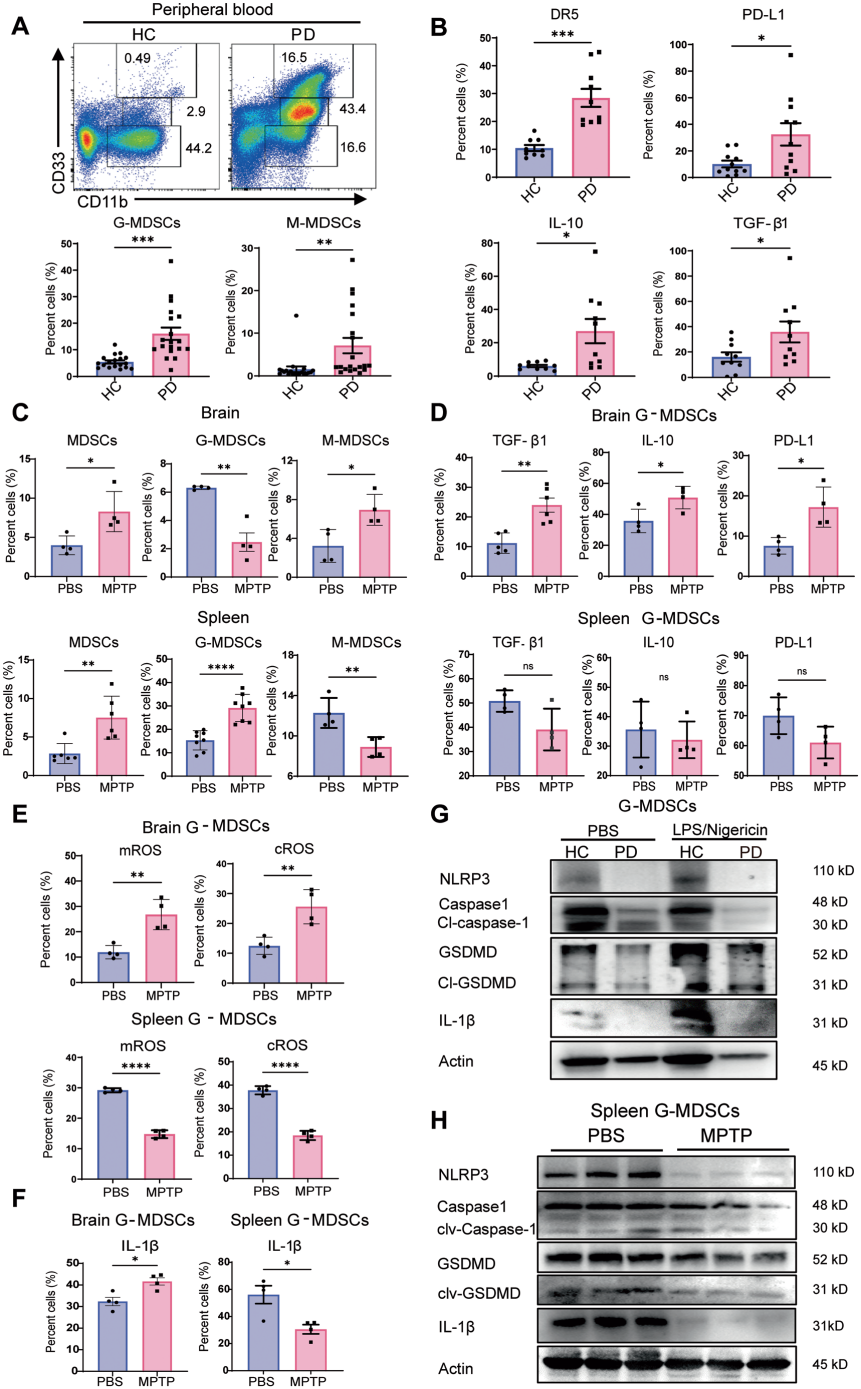
Distribution of G‐MDSCs in PD individuals. (A) Peripheral blood M‐MDSCs were gated as HLA‐DR^−^CD11b^+^CD33^high^ cells, and G‐MDSCs gated as HLA‐DR^−^CD11b^+^CD33^low^ cells (*n* = 19). (B) Expressions of DR5, PD‐L1, IL‐10, and TGF‐β1 in G‐MDSCs detected by flow cytometry (*n* = 10). (C) Variations of G‐MDSCs and M‐MDSCs in the brain and spleen of MPTP‐treated mice (*n* = 4). (D–F) PD‐L1, IL‐10, TGF‐β1, cROS, mROS, and IL‐1β of murine G‐MDSCs (*n* = 4). (G) G‐MDSCs were primed with LPS (100 ng/mL, 4 h) and subsequently stimulated with Nigericin (20 μM, 1 h). Protein levels of NLRP3, full‐length and cleaved GSDMD, cleaved caspase‐1, and IL‐1β in G‐MDSCs of peripheral blood. (H) Variations of NLRP3, caspase 1, GSDMD, and IL‐1β in splenic G‐MDSCs of MPTP‐treated mice. All animal experiments were repeated twice. Ns, no significance; *p < 0.05; **p < 0.01; ***p < 0.001; ****p < 0.0001.

Firstly, the distributions of G‐MDSCs (HLA‐DR^−^CD11b^+^CD33^low^) and M‐MDSCs (HLA‐DR^−^CD11b^+^CD33^high^) in the peripheral blood of patients with PD and healthy controls were examined. The patients with PD had increased G‐MDSCs and M‐MDSCs in their blood, with a pronounced increase of G‐MDSCs (Figure 1A). These MDSCs of patients with PD showed an enhancement in the expression levels of DR5, PD‐L1, IL‐10, and TGF‐β1 (Figure 1B). Secondly, the distributions of G‐MDSCs/M‐MDSCs in the brain and spleen between MPTP‐induced PD and normal mice were compared. Increased MDSCs were seen in the brain and spleen of PD mice. However, G‐MDSCs increased in the spleen and decreased in the brain of PD mice. Meanwhile, M‐MDSCs decreased in the spleen but increased in the brain (Figure 1C and Figure S1A,B). Considering the plasticity role of M‐MDSCs in inflammatory conditions [12, 13], the present study focused on G‐MDSCs in PD progression.

Although G‐MDSCs decreased in the brain of PD mice, these cells had high expression levels of TGF‐β1, IL‐10, and PD‐L1. Meanwhile, the splenic G‐MDSCs had no changes in the above effector molecules (Figure 1D). Increased mitochondrial ROS (mROS) is a key event for NLRP3 inflammasome activation to provide secondary signaling [14]. The brain and splenic G‐MDSCs had increased and decreased levels of mROS and cellular ROS (cROS), respectively (Figure 1E). Meanwhile, the cellular IL‐1β in the brain and splenic G‐MDSCs increased and decreased, respectively (Figure 1F). The G‐MDSCs in the peripheral blood of patients with PD demonstrated a decrease in the expression levels of NLRP3, cleaved‐caspase 1, cleaved‐GSDMD, and IL‐1β (Figure 1G and Figure S1C). Sufficient G‐MDSCs could not be obtained in the brain for Western blot analysis. However, the splenic G‐MDSCs had low expression levels of NLRP3, cleaved caspase 1, IL‐1β, and cleaved GSDMD (Figure 1H). Thus, increased peripheral G‐MDSCs with low GSDMD activation was demonstrated in PD individuals.

### 3.2 Amelioration of MPTP‐Induced PD in GSDMD ^−/−^ Mice by G‐MDSCs

**FIGURE 2:**
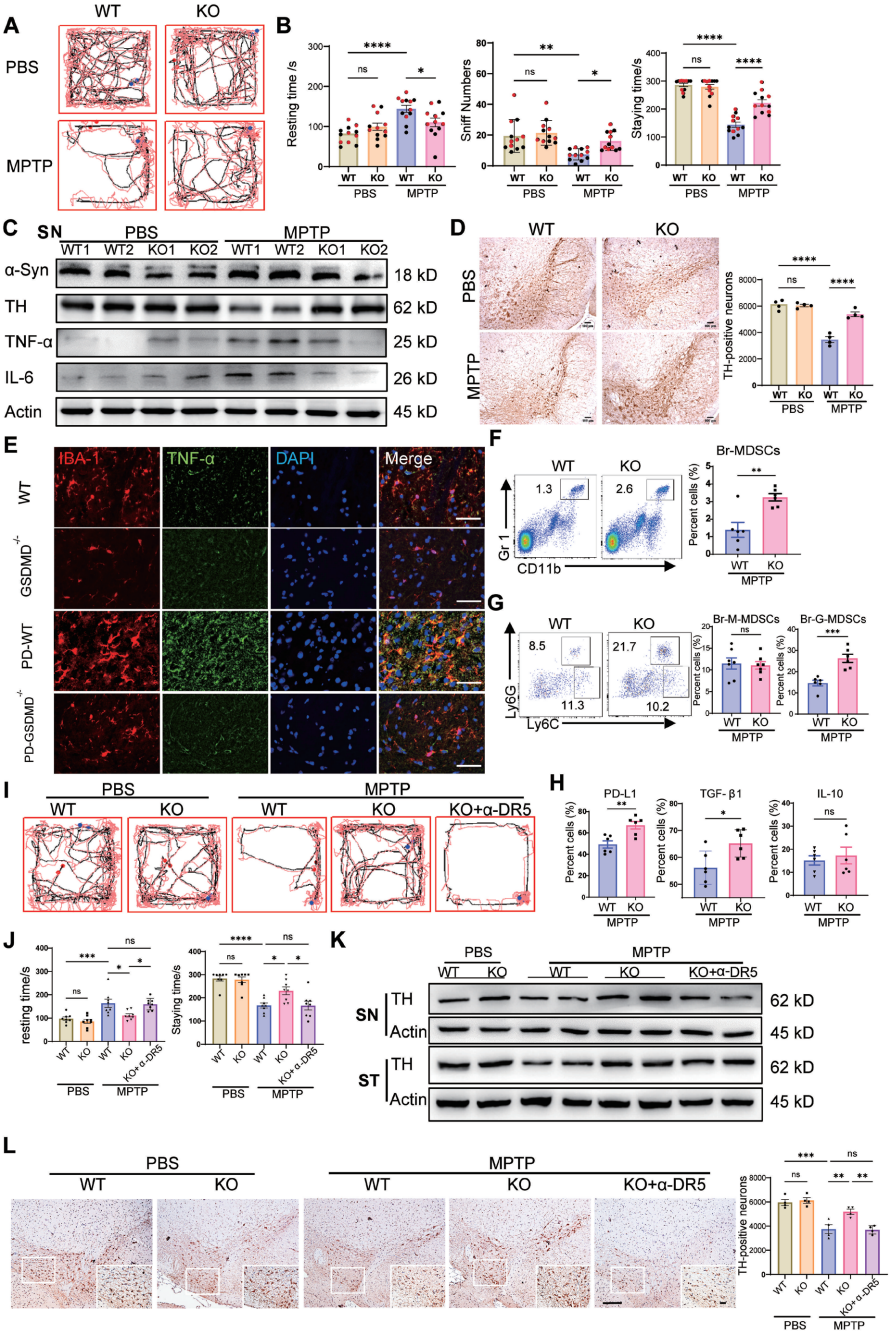
Relieved PD symptoms in GSDMD^−/−^ mice. (A, B) Action trajectory, immobility time, sniff numbers in open field tests and staying time in rotarod tests (*n* = 12, red for females, black for males). (C) Western‐blot analysis of α‐synuclein, TH, TNF‐α, and IL‐6 in substantia nigra of WT or KO mice treated with MPTP. (D) TH expression of SN detected by IHC. (E) Co‐staining of IBA‐1 with TNF‐α in SN by IF, scale bar = 20 μm. (F, G) Detection of G‐MDSCs in MPTP‐treated WT/KO mice (*n* = 6). (H) Brain PD‐L1^+^, IL‐10^+^, and TGF‐β1^+^ G‐MDSCs detected by flow cytometry (*n* = 6). (I, J) Behavioral experiments of mice depleted of MDSCs by α‐DR5 (*n* = 8). (K, L) Depletion of MDSCs restored the decrease of TH level in SN of KO mice as measured by western blot and IHC, scale bar = 100 μm. All animal experiments were performed at least twice. Ns, no significance; *p < 0.05; **p < 0.01; ***p < 0.001; ****p < 0.0001.

GSDMD‐KO and wild‐type (WT) mice of both sexes were treated with MPTP, and their behavioral changes were analyzed. The GSDMD^−/−^ mice exhibited alleviated behavioral disorders in comparison with WT mice, as demonstrated by action trajectory (Figure 2A), immobility time, and sniff numbers in open‐field tests (Figure 2B). The staying time in rotarod tests was higher in KO mice than in WT mice (Figure 2B). With MPTP treatment, the tyrosine hydroxylase (TH) level in the substantia nigra and corpus striatum of KO mice decreased compared with that in WT mice (Figure 2C,D and Figure S2A,B). The KO mice demonstrated decreased productions of α‐synuclein, TNF‐α, and IL‐6 in the substantia nigra and corpus striatum (Figure 2C and Figure S2A). The variated TNF‐α of microglia (IBA1) was verified in the substantia nigra (Figure 2E) and striatum (Figure S2C) of MPTP‐treated KO mice by IHC. Considering no obvious changes were observed in the MDSCs of caspase1^−/−^ mice, they had similar behavioral changes in the open‐field test with the WT mice (Figure S2D).

The distributions of MDSCs in the brain were further examined. As expected, the brain MDSCs in GSDMD^−/−^ mice remarkably enhanced (Figure 2F), with an obvious increase in G‐MDSCs (Figure 2G). These brain G‐MDSCs had high expression levels of PD‐L1 and TGF‐β1 (Figure 2H) with no variation in IL‐10. When the MDSCs were depleted with DR5 antibody in GSDMD^−/−^ mice, the mobility of these mice in the open‐field test sharply decreased, similar to that of MPTP‐treated WT mice (Figure 2I,J). After MDSCs were depleted, the average speed of GSDMD^−/−^ mice in rotarod tests recovered (Figure 2J). Finally, the TH in the substantia nigra and striatum was profoundly downregulated in KO mice treated with DR5 antibody, as detected by Western blot (Figure 2K) and immunohistochemistry (Figure 2L and Figure S2E). Collectively, the attenuation of MPTP‐induced PD in GSDMD^−/−^ mice was dependent on G‐MDSCs.

### 3.3 Alleviation of MPTP‐Induced PD in GSDMD^flox^ ^/flox^‐S100A8^cre^ Mice

**FIGURE 3:**
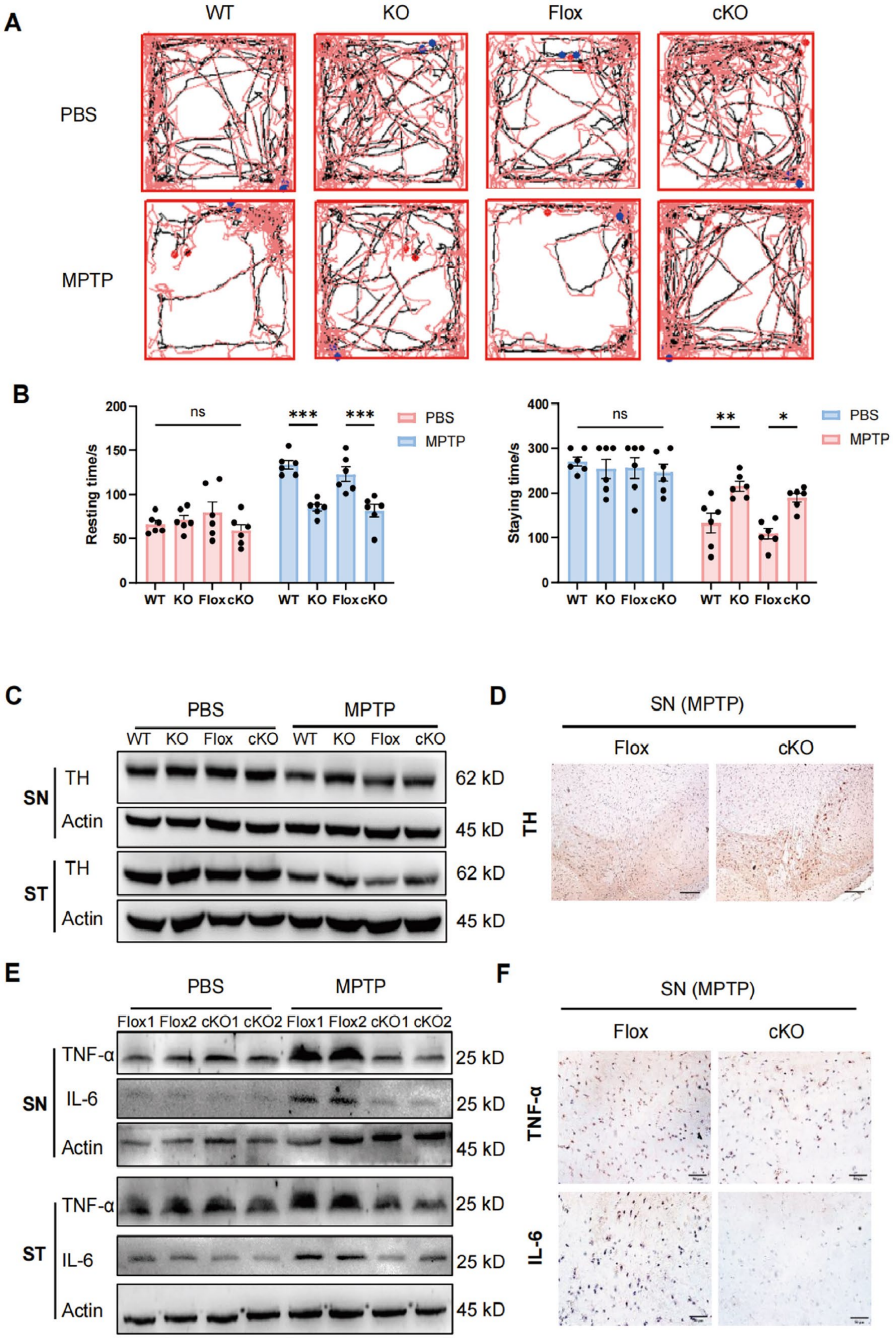
Amelioration of PD in GSDMD‐cKO mice. (A, B) Behavioral experiments of cKO mice (*n* = 6). (C) Variations of TH in SN of WT, KO, Flox, and cKO mice detected by western blot. (D) TH variations in SN between Flox and cKO mice measured by IHC, scale bar = 100 μm. (E, F) Variations of TNF‐α and IL‐6 in SN between Flox and cKO mice. All experiments were performed at least twice. Ns, no significance; *p < 0.05; **p < 0.01; ***p < 0.001.

Conditional knockout (cKO) mice were cross‐generated with a specific deficiency of GSDMD in myeloid cells (GSDMD^flox/flox^‐S100A8^cre^) by using S100A8^cre^ mice. Similar to KO mice, the cKO mice demonstrated moderate severity of PD after MPTP treatment, as supported by action trajectory (Figure 3A) and immobility time in open‐field tests (Figure 3B) and staying time in rotarod tests (Figure 3B). Compared with the GSDMD^flox^ mice, the MPTP‐treated cKO mice had enhanced TH (Figure 3C,D and Figure S3A) and decreased TNF‐α and IL‐6 (Figure 3E) in the substantia nigra and striatum. The staining intensity of TNF‐α and IL‐6 in the substantia nigra and striatum was substantially lighter in cKO mice than in GSDMD^flox^ mice (Figure 3F and Figure S3B). The results showed that GSDMD^−/−^ MDSCs exert a protective role against PD progression.

### 3.4 Adoptive Transfer of GSDMD ^−/−^ G‐MDSCs Limiting PD Progression

**FIGURE 4:**
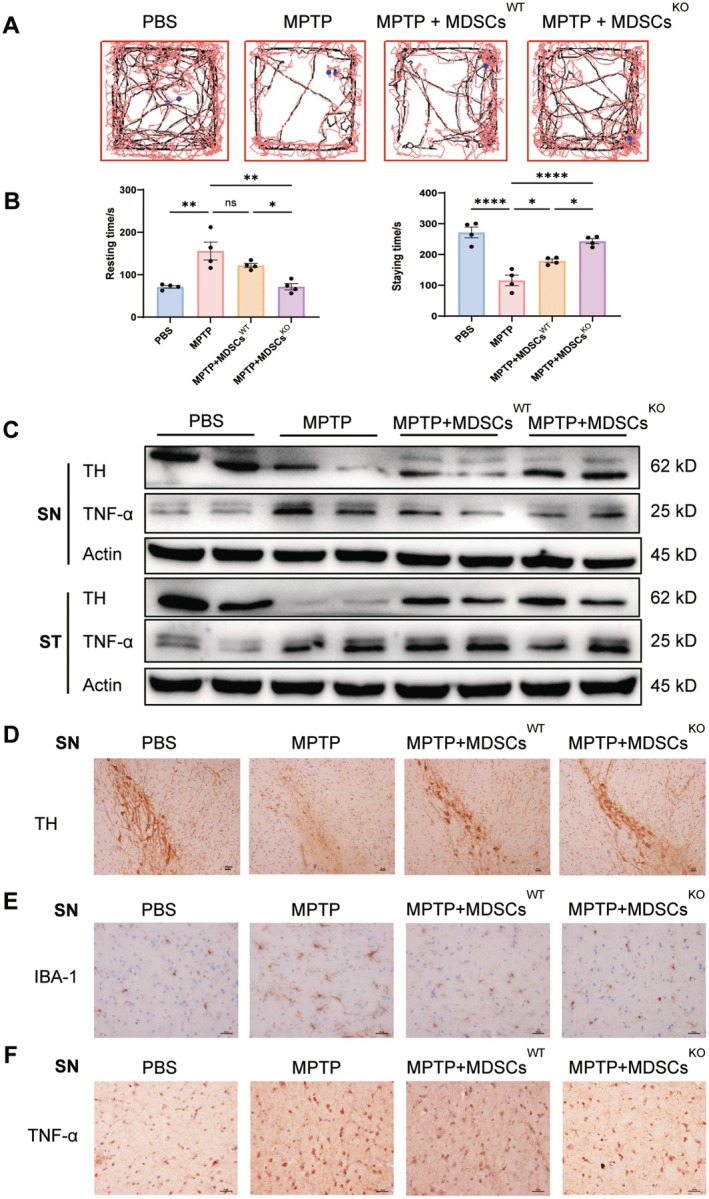
Infusion of GSDMD^−/−^G‐MDSCs protects against MPTP‐induced PD. (A, B) Behavioral experiments of MPTP‐treated mice with infusion of MDSCs (*n* = 4). (C) Western‐blot analysis of TH and TNF‐α in SN of the above mice. (D–F) Analysis of TH, IBA‐1, and TNF‐α in SN of the above mice by IHC. All animal experiments were performed at least twice. Ns, no significance; *p < 0.05; **p < 0.01; ***p < 0.001; ****p < 0.0001.

The CD11b^+^ GR1^+^ cells from WT and KO mice were isolated and adoptively transferred into MPTP‐treated WT mice. The infusion of either normal or GSDMD‐deficient MDSCs was able to enhance the mobility of PD mice, and the transfer of MDSCs^KO^ had better improvements than that of MDSCs^WT^ (Figure 4A). The resting time in open‐field tests and the staying time in rotarod tests confirmed that the infusion of MDSCs^KO^ is more effective in ameliorating PD symptoms (Figure 4B). The better alleviation against PD by MDSCs^KO^ was further supported by less loss of TH and less production of TNF‐α in the substantia nigra and striatum (Figure 4C), as detected by Western blot. Immunohistology analysis displayed enhanced TH recovery (Figure 4D and Figure S4A), decreased amoeba morphology (Figure 4E and Figure S4B), and low TNF‐α staining (Figure 4F and Figure S4C) in the substantia nigra and striatum (Figure S4A–C) by the transfer of MDSCs^KO^. These results demonstrated that the infusion of GSDMD^−/−^ MDSCs can be beneficial to suppress MPTP‐induced PD.

### 3.5 GSDMD ^−/−^ G‐MDSC Inhibition of Inflammatory Activity of Microglia Ex Vivo

**FIGURE 5:**
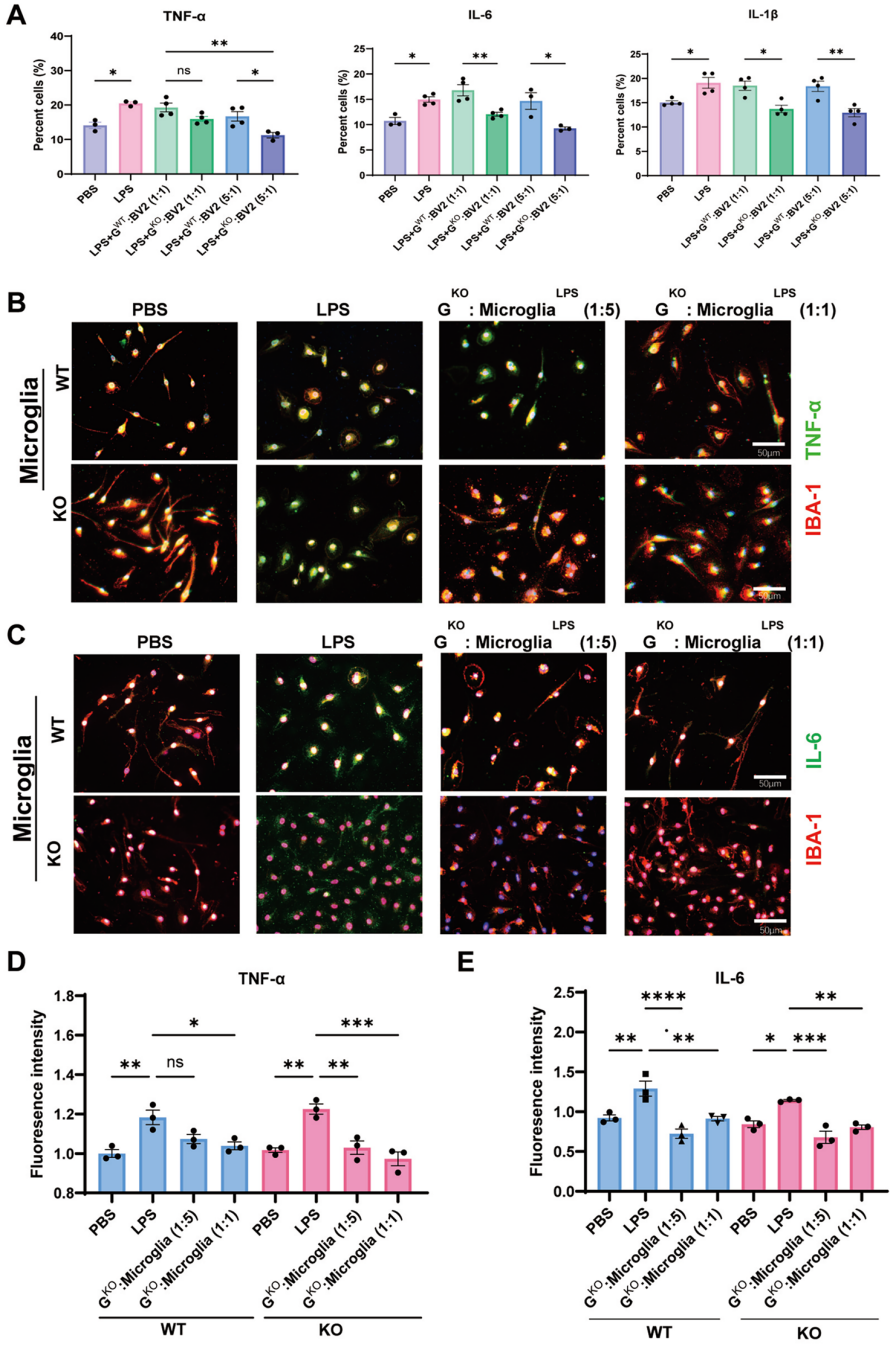
GSDMD^−/−^G‐MDSCs directly inhibit activities of LPS‐stimulated microglia. (A) Flow‐cytometric analysis of TNF‐α, IL‐6, and IL‐1β of BV2 cells as cocultured with G‐MDSCs (*n* = 3). (B, C) Co‐staining of TNF‐α or IL‐6 with IBA‐1 detected by IF. (D, E) Statistics of fluorescence density (*n* = 3). The cell experiments were repeated at least twice. Ns, no significance; *p < 0.05; **p < 0.01; ***p < 0.001; ****p < 0.0001.

Considering that PD is a result of chronic inflammation [15], overactivated microglia have detrimental effects on dopaminergic neurons [16, 17]. Whether the production of pro‐inflammatory cytokines by LPS‐stimulated BV2 cells (a microglia cell line) was affected by G‐MDSCs was analyzed. When G‐MDSCs^KO^ were cocultured with BV2 cells, the secretions of TNF‐α, IL‐6, and IL‐1β by BV2 cells decreased, particularly at the ratio of 5:1 (Figure 5A and Figure S5A–C). Then, primary microglial cells were isolated either from WT or KO mice. These microglia were firstly stimulated by LPS and then cocultured with GSDMD^−/−^ G‐MDSCs. As expected, the GSDMD^−/−^ G‐MDSCs efficiently suppressed the TNF‐α (Figure 5B,D) and IL‐6 (Figure 5C,E) production of microglia, either from the WT or KO mice. Thus, GSDMD^−/−^ G‐MDSCs exert profoundly suppressive activity to inhibit the inflammatory activity of LPS‐stimulated microglia.

### 3.6 ACT001 Protection Against PD via Upregulating G‐MDSC Activities

**FIGURE 6:**
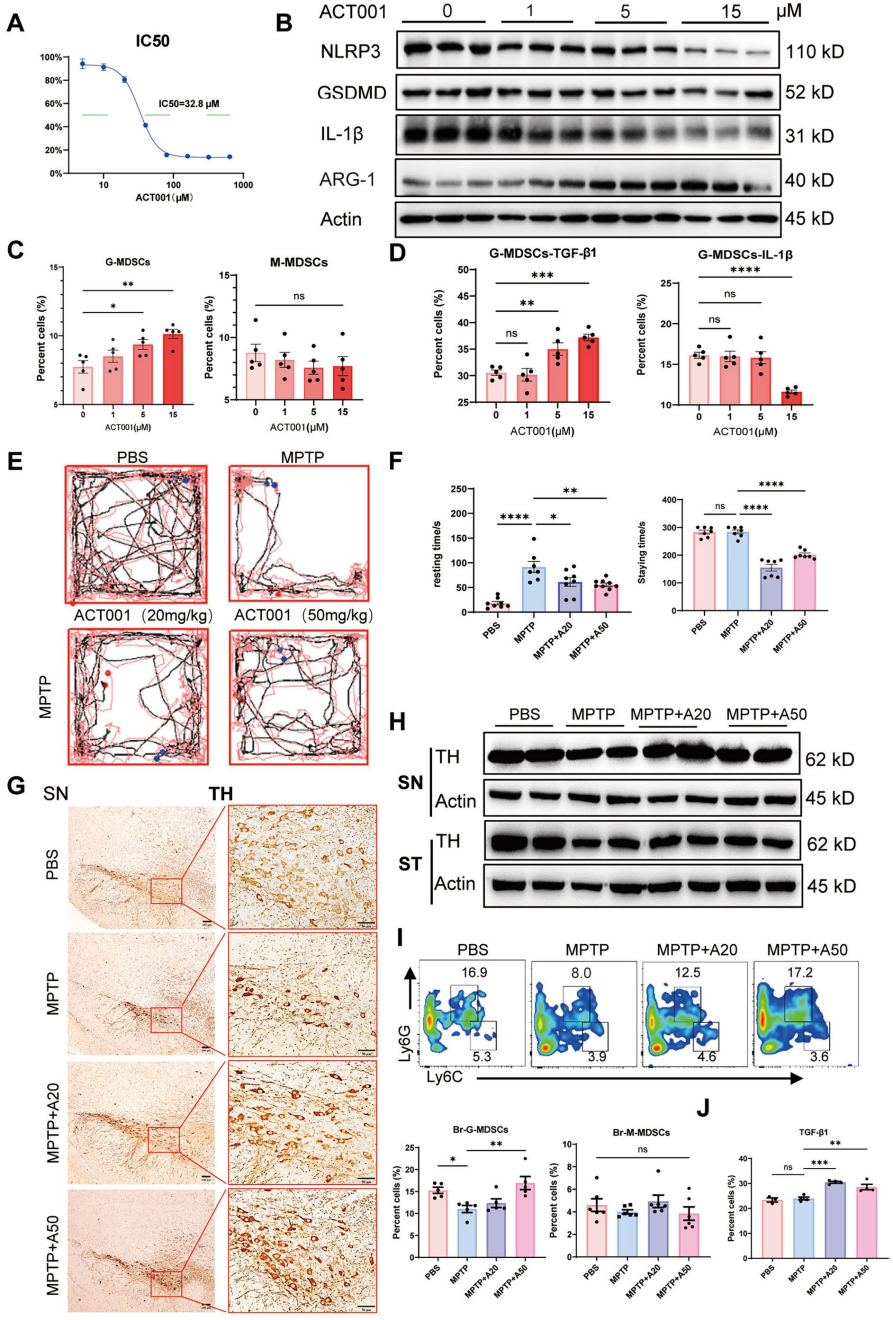
ACT001 inhibits PD progression involved with G‐MDSCs. (A) The IC50 of ACT001 on MDSCs was determined by CCK‐8 assay. (B) Variations of NLRP3, GSDMD, IL‐1β, and ARG1 in MDSCs treated with a variety of doses of ACT001. (C, D) Frequencies of G‐MDSCs and their expressions of TGF‐β1 and IL‐1β detected by flow cytometry (*n* = 5). (E, F) Behavioral experiments of MPTP‐treated mice administered with ACT001 (*n* = 7). (G, H) Changes of TH levels in SN and ST of ACT001‐treated PD mice. (I) Brain G/M‐MDSCs of mice by the treatment of ACT001. (J) TGF‐β1 production of brain G‐MDSCs in ACT001‐treated PD mice (*n* = 6). All experiments were performed at least twice. Ns, no significance; *p < 0.05; **p < 0.01; ***p < 0.001; ****p < 0.0001.

As a fumarate salt form of dimethylaminomicheliolide (DMAMCL), ACT001 is able to suppress MPTP‐induced α‐synuclein accumulation, microglial and astrocyte activation, and inhibit dopaminergic neurodegeneration via downregulating NLRP3 inflammasome‐induced inflammation [18, 19]. Whether ACT001 treatment affects G‐MDSC activity was then investigated. The half maximal inhibitory concentration (IC50) of ACT001 acting on G‐MDSCs was determined (32.8 μM, Figure 6A). The G‐MDSCs showed a sharp decrease in the expression levels of NLRP3, GSDMD, and IL‐1β and an increase in ARG1 production in a dose‐dependent manner after ACT001 treatment (Figure 6B). ACT001 directly promoted the induction of G‐MDSCs but not M‐MDSCs in vitro (Figure 6C and S6A). Decreased IL‐1β and increased TGF‐β1 were shown in these ACT001‐treated G‐MDSCs (Figure 6D).

The improvement of ACT001 on mice with MPTP‐induced PD was confirmed by behavioral experiments. With the treatment of ACT001, the PD mice showed increased mobility and balance ability, as identified by the open‐field or rotating rod experiment (Figure 6E,F). The histological analysis of substantia nigra and striatum demonstrated enhanced staining of TH in PD mice with ACT001 injection (Figure 6G and Figure S6B). The increased TH level in the substantia nigra and striatum of ACT001‐treated PD mice was further confirmed by Western blot analysis (Figure 6H). Finally, the ACT001‐treated PD mice had increased G‐MDSCs in the brain and no changes in M‐MDSCs (Figure 6I). These brain G‐MDSCs were able to produce TGF‐β1, which could perform immunosuppressive effects in vivo (Figure 6J). Thus, the amelioration of ACT001 in PD should involve the upregulation of G‐MDSC bioactivities.

## 4 Discussion

MDSCs have been shown to be involved in PD progression [8, 9]. Enhancing the immune regulatory activity of MDSCs can theoretically inhibit the progression of this inflammation‐induced chronic disease. Here, the G‐MDSCs increased in the periphery of patients and mice with PD and decreased in the CNS of PD mice. The peripheral G‐MDSCs showed a decrease in NLRP3/GSDMD inflammasome activation. The mice with ubiquitous deficiency of GSDMD or myeloid (S100A8^+^) cell deficiency of GSDMD demonstrated relieved PD symptoms. The amelioration of PD in GSDMD‐deficient mice disappeared after the MDSCs were pre‐depleted with DR5 antibody. Adoptive transfer of GSDMD‐deficient G‐MDSCs can directly repress PD progression. These GSDMD‐deficient G‐MDSCs efficiently inhibited the pro‐inflammatory activity of microglial cells in vitro. Finally, ACT001, as an inhibitor against NLRP3 activation, induced G‐MDSCs ex vivo and exerted a protective effect against PD with the enhanced presence of G‐MDSCs in the brain. Altogether, adoptive transfer of GSDMD‐deficient G‐MDSCs or inhibition of GSDMD activation of MDSCs in vivo could be exploited to treat inflammation‐associated diseases.

The patients with PD had increased G‐MDSCs in their peripheral blood. The G‐MDSCs in the spleen and brain of PD mice increased and decreased, respectively (Figure 1). Decreased G‐MDSCs in the brain could be predicted in patients with PD by considering the BBB of the human body. Although low numbers of G‐MDSCs were present in the brain of MPTP‐induced PD mice, these cells had a high production of TGF‐β1, IL‐10, and PD‐L1 (Figure 1), suggesting that brain G‐MDSCs exert immunosuppressive activity. The decreased NLRP3/GSDMD activation (Figure 1) with enhanced G‐MDSCs in the spleen confirmed that inhibiting GSDMD activation can promote the induction of G‐MDSCs. Meanwhile, the brain G‐MDSCs had increased mROS production and cytoplasmic IL‐1β (Figure 1) under the inflammatory microenvironment in the CNS of PD mice, indicating high activation of GSDMD. Thus, high activation of GSDMD indicated low induction of G‐MDSCs under local inflammation.

Building on the observation that GSDMD deficiency suppressed neuroinflammation in a mouse model of PD [20], our study extends these findings by characterizing the immunomodulatory roles of GSDMD‐deficient G‐MDSCs in the context of PD. The adoptive transfer of GSDMD^−/−^ G‐MDSCs by tail vein could efficiently suppress the progression of PD. In spite of the increased BBB permeability in MPTP‐treated mice, the recruitment of these infused G‐MDSCs into the CNS was not observed. However, the immune‐inhibitory effects of GSDMD^−/−^ G‐MDSCs could simultaneously attenuate peripheral inflammation. Reducing peripheral inflammation can theoretically alleviate the degree of inflammation in the CNS [21, 22]. Besides, TGF‐β1 derived from G‐MDSCs may cross the BBB with increased permeability to exert immune regulatory effects and inhibit local inflammation in individuals with PD [23].

Microglia, the principal immune cells in the CNS, are critically involved in the pathogenesis of neurodegenerative diseases [24]. Evidence suggested that peripheral infections could trigger pyroptosis in peripheral myeloid cells, subsequently initiating immune training in microglia. This process was implicated in promoting neuroinflammation and eliciting PD‐like symptoms [25]. Although our study did not directly assess the immune memory of microglia, the experimental results suggested that GSDMD‐deficient G‐MDSCs may possess the capacity to modulate microglial immune memory. Specifically, we observed that GSDMD^−/−^ G‐MDSCs exhibited significantly higher expression levels of anti‐inflammatory cytokines, including TGF‐β1 and IL‐10, and demonstrably lower expression levels of pro‐inflammatory cytokines, such as IL‐1β and TNF‐α. These cytokines were known regulators of microglial immune status and were implicated in the formation of immune memory through their influence on key transcription factors, including NF‐κB and STAT3, and by altering microglial gene expression profiles [26].

The molecular mechanisms of G‐MDSCs induction by GSDMD loss were previously elucidated by the authors' group. Due to the presence of GSDMD on the mitochondrial membrane, GSDMD deficiency not only reduced cell apoptosis but also led to reduced mitochondrial DNA release into the cytoplasm [27, 28]. cGAS/Sting activation is thereby inhibited, leading to the decreased expression of IRF7/8 and the promoted differentiation of myeloid cells into G‐MDSCs. G‐MDSCs could be induced by disulfiram due to its direct inhibitory effects against GSDMD activation [29]. However, the use of disulfiram is severally influenced by alcohol drinking. The therapeutic promise of GSDMD inhibition hinges on effectively balancing its benefits against potential risks, a balance intrinsically tied to its impact on the local cellular microenvironment. As demonstrated through our studies, a successful therapeutic strategy must involve context‐specific modulation, targeting GSDMD inhibition to pathological microenvironments rather than systemic suppression.

ACT001 is projected for therapeutic application in gliomas and PD, attributed to its minimal adverse effects and significant BBB permeability [30]. Mechanistically, ACT001 attenuates neuroinflammation by suppressing the NLRP3 inflammasome pathway in microglia, achieved through inhibition of AKT phosphorylation and subsequent blockade of NF‐κB nuclear translocation. ACT001 treatment could exert dual protection against PD: direct inhibition of inflammasome activation in pro‐inflammatory cells [18, 19], such as microglia, and induction of immune‐suppressive cells (MDSCs). Consequently, short‐term ACT001 administration may suppress neuroinflammation; however, the possibility of unexpected consequences or side effects with chronic administration, especially in autoimmune tendencies, requires further clinical validation [31].

## 5 Conclusion

This study showed that G‐MDSCs are involved in PD progression. The MPTP‐induced PD in GSDMD^−/−^ mice was ameliorated with increased G‐MDSCs. The mice with cKO GSDMD in S100A8^+^ myeloid cells showed attenuated PD symptoms. The adoptive transfer of GSDMD^−/−^ G‐MDSCs efficiently relieved PD progression. The protection of ACT001 in PD mice was accompanied by increased G‐MDSCs in the brain. Thus, methods to disturb GSDMD activation to enhance the immunosuppressive activity of G‐MDSCs could be exploited in the therapy of PD.

## Author Contributions

**Qi Wu:** writing – original draft, validation, investigation, visualization. **Fangzhou Liu:** validation, investigation, data curation. **Min Gu:** validation, visualization. **Junjie Wei:** validation, visualization. **Guotao Lu:** resources, supervision, funding acquisition. **Li Qian:** resources, supervision. **Xiaobo Li:** writing – review and editing, project administration, conceptualization. **Weijuan Gong:** writing – review and editing, project administration, funding acquisition.

## Ethics Statement

All blood samples were obtained according to the ethical requirements of Subei People's Hospital, Jiangsu Province (No. 2024ky080). All animal work was approved by the Ethics Committee of the Medical College of Yangzhou University (YXYLL‐2020‐67).

## Conflicts of Interest

The authors declare no conflicts of interest.

## Supporting information


**Figure S1:** A representative FCM dot plot for MDSCs in the brain and spleen of PD mice. (A) Brain MDSCs were gated as CD11b + Gr‐1 + cells, G‐MDSCs gated as CD11b+ Ly6ClowLy6G+ cells, and M‐MDSCs gated as CD11b+ Ly6ChighLy6G−. (B) Spleen MDSCs and their typing. (C) Quantification of NLRP3, caspase‐1, cleaved caspase‐1, GSDMD, cleaved GSDMD and IL‐1β levels in G‐MDSCs from peripheral blood.
**Figure S2:** Variations of TH levels in ST of MPTP‐treated mice. (A) Western‐blot analysis of α‐synuclein, TH, TNF‐α, and IL‐6 in striatum of WT or KO mice treated with MPTP. (B) Staining of TH in ST of WT or KO mice before and after the MPTP treatment. (C) Co‐staining of IBA‐1 with TNF‐α in ST of above mice. (D) Action trajectory of open field tests was documented among WT, GSDMD^−/−^, and Caspase 1^−/−^ mice. (E) The TH variation of KO mice depleted with MDSCs by α‐DR5.
**Figure S3:** Histological changes in ST of GSDMD‐Flox and GSDMD‐cKO mice. (A) Staining of ST. (B) Staining of TNF‐α and IL‐6.
**Figure S4:** Histological changes in ST of MPTP‐treated mice infused with MDSCs. Staining of TH (A), IBA‐1 (B), and TNF‐α (C).
**Figure S5:** Representative results of TNF‐α (A), IL‐6 (B), and IL‐1β (C) production by BV2 cells detected by flow cytometry.
**Figure S6:** ACT001 promotes G‐MDSCs to inhibit PD progression. (A) Representative flow‐cytometric results of G/M‐MDSCs induced by ACT001 ex vivo. (B) Staining of TH in ST of PD mice treated with ACT001.

**Table S1:** Clinical characteristics of PD patients and healthy controls (HC).

## Data Availability

The data that support the findings of this study are available from the corresponding author upon reasonable request.
