# Correction to “GSDMD‐Deficient G‐MDSCs Exert Profoundly Suppressive Activity to Relieve MPTP‐Induced Parkinson's Disease”

**DOI:** 10.1002/cns.70945

**Published:** 2026-08-20

Q. Wu, F. Liu, M. Gu, et al., “GSDMD‐Deficient G‐MDSCs Exert Profoundly Suppressive Activity to Relieve MPTP‐Induced Parkinson's Disease,” *CNS Neuroscience & Therapeutics* 31, no. 10 (2025): e70626, https://doi.org/10.1111/cns.70626.

In the Methods section of the Abstract, the text “Flow cytometry and Western blot analyzed G/M‐MDSCs in 37 PD patients and 21 controls.” was incorrect.

This should have read: “Flow cytometry was used to analyze G/M‐MDSCs in peripheral blood from 19 of the 37 enrolled PD patients, while samples from the remaining patients were used for Western blotting.” The legend of Figure 1A has also been corrected to accurately reflect the sample allocation.

In Figure 3D, the Flox in SN (MPTP) panel was inadvertently duplicated from Figure 2L (KO+αDR5 in SN; MPTP). Figure 2L is correct as published. The correct original image for Figure 3D is provided below.

In addition, the statistical bar graph in Figure 6F (rotarod test) was incorrectly plotted, and the corrected figure is provided below.

The authors confirm that all experimental results and corresponding conclusions in the paper remain unaffected.

The authors apologize for these errors.

Corrected Figure 1

**Figure:**
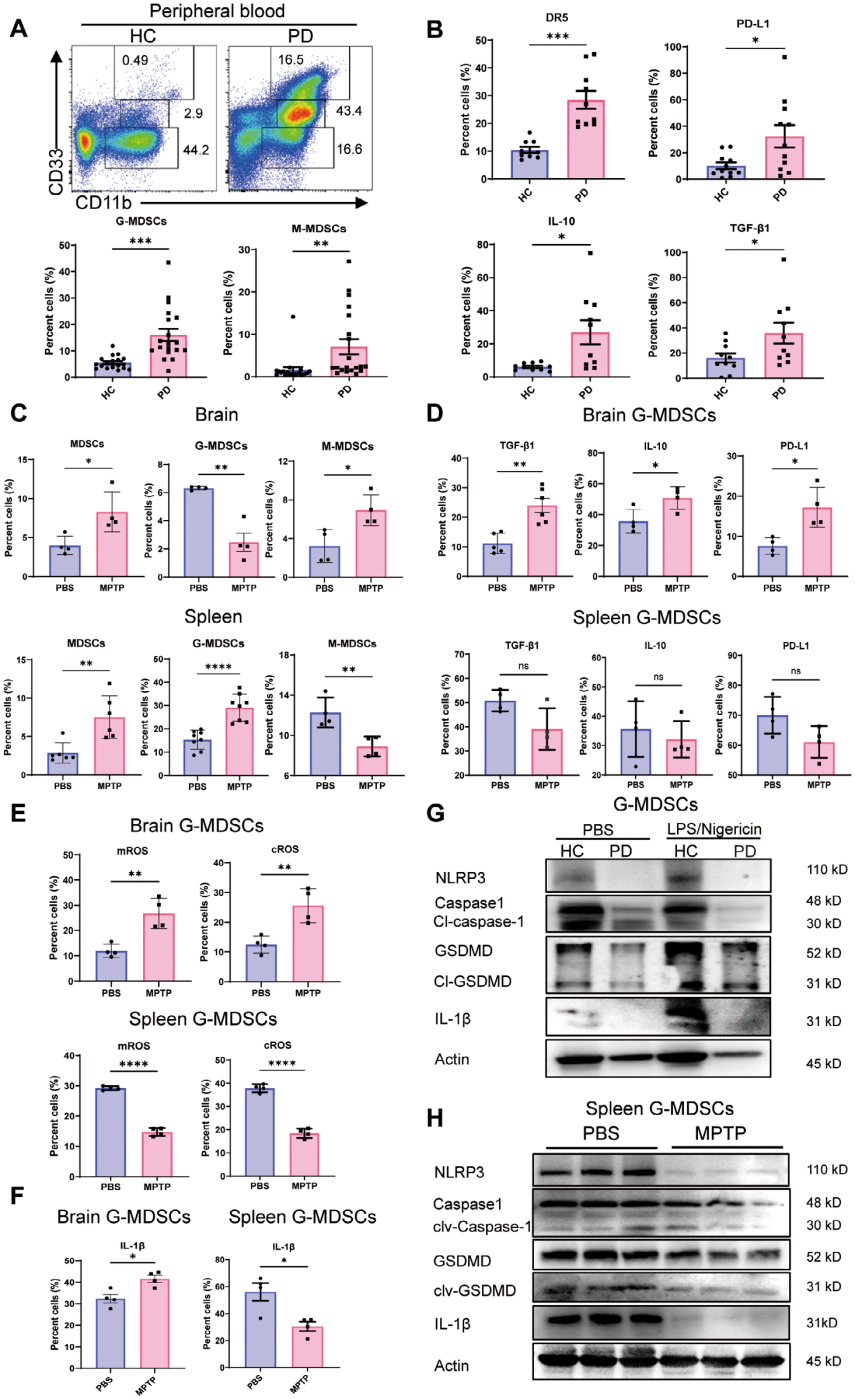
**FIGURE 1**Distribution of G‐MDSCs in PD individuals. (A) Frequencies of circulating G‐MDSCs (gated as HLA‐DR^−^CD11b^+^CD33ˡ°ʷ cells) and M‐MDSCs (gated as HLA‐DR^−^CD11b^+^CD33ʰⁱᵍʰ) in peripheral blood from 19 randomly selected PD patients and 19 healthy controls. (B) Expression levels of DR5, PD‐L1, IL‐10, and TGF‐β1 in G‐MDSCs from 10 PD patients and 10 healthy controls (randomly selected from the 19 PD patients and 19 healthy controls in A). (C) Variations of G‐MDSCs and M‐MDSCs in brain and spleen of MPTP‐treated mice (*n* = 4). (D‐F) PD‐L1, IL‐10, TGF‐β1, cROS, mROS, and IL‐1β of murine G‐MDSCs (*n* = 4). (G) G‐MDSCs were primed with LPS (100 ng/mL, 4 h) and subsequently stimulated with Nigericin (20 μM, 1 h). Protein levels of NLRP3, full‐length and cleaved GSDMD, cleaved caspase‐1, and IL‐1β in G‐MDSCs of peripheral blood. (H) Variations of NLRP3, caspase 1, GSDMD, IL‐1β in splenic G‐MDSCs of MPTP‐treated mice. All animal experiments were repeated twice. Ns, no significance; *p < 0.05; **p < 0.01; ***p < 0.001.

Corrected Figure 3

**Figure:**
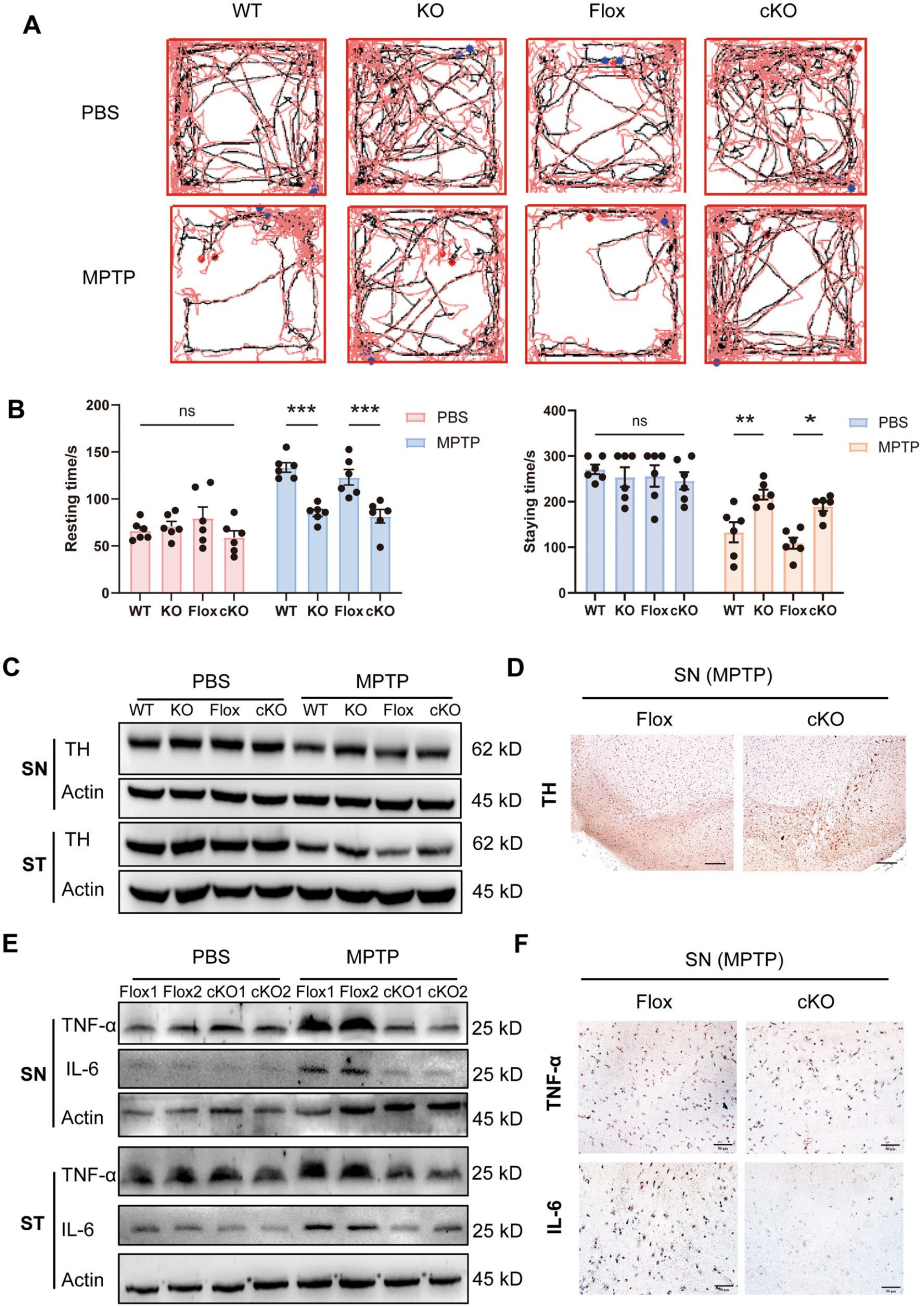
**FIGURE 3**Amelioration of PD in GSDMD‐cKO mice. (A, B) Behavioral experiments of cKO mice (*n* = 6). (C) Variations of TH in SN of WT, KO, Flox, and cKO mice detected by western blot. (D) TH variations in SN between Flox and cKO mice measured by IHC, scale bar = 100 μm. (E, F) Variations of TNF‐α and IL‐6 in SN between Flox and cKO mice. All experiments were performed at least twice. Ns, no significance; *p < 0.05; **p < 0.01; ***p < 0.001.

Corrected Figure 6

**Figure:**
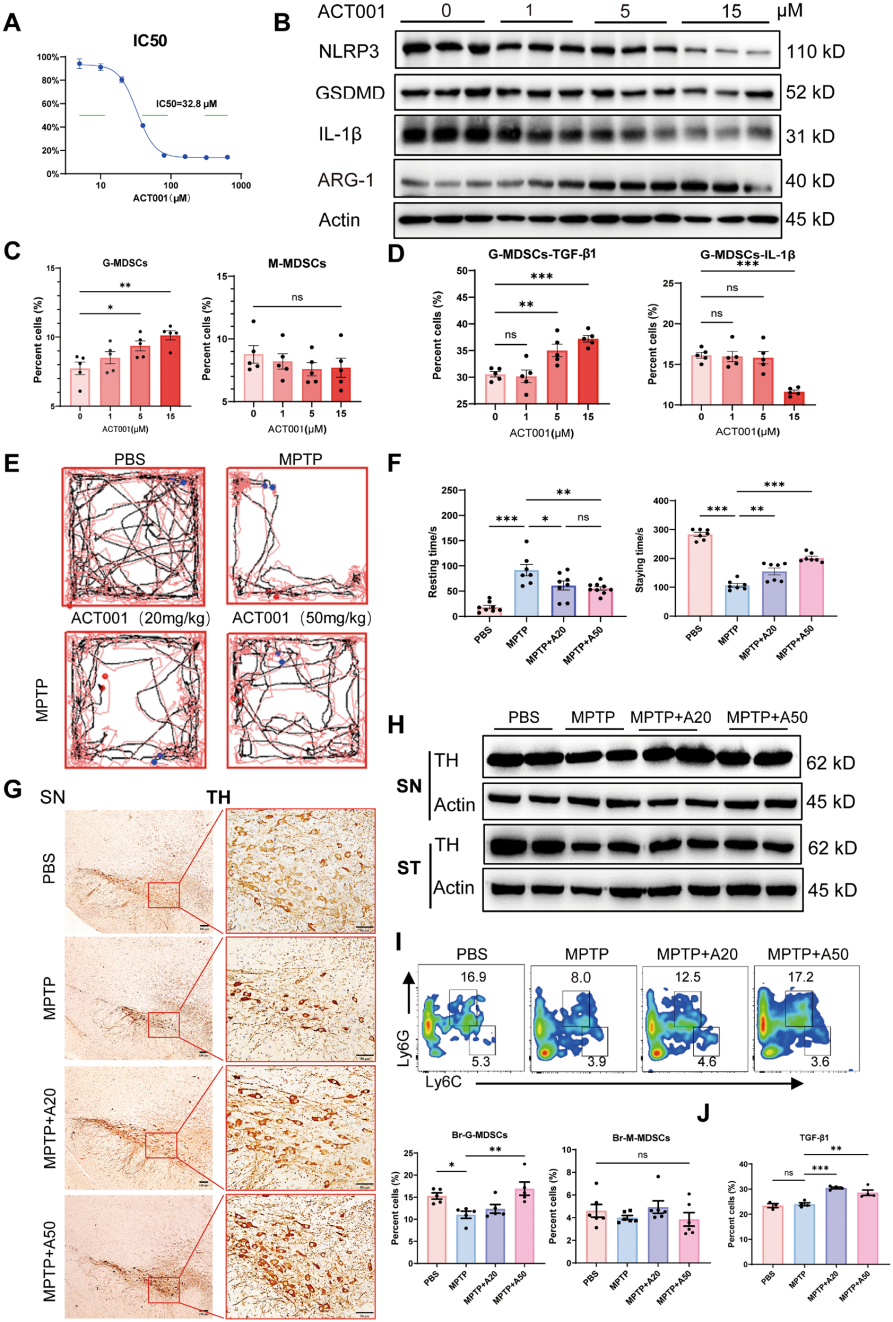
**FIGURE 6**ACT001 inhibits PD progression involved with G‐MDSCs. (A) The IC50 of ACT001 on MDSCs was determined by CCK‐8 assay. (B) Variations of NLRP3, GSDMD, IL‐1β, and ARG1 in MDSCs treated with a variety of doses of ACT001. (C, D) Frequencies of G‐MDSCs and their expressions of TGF‐β1 and IL‐1β detected by flow cytometry (*n* = 5). (E, F) Behavioral experiments of MPTP‐treated mice administrated with ACT001 (*n* = 7). (G, H) Changes of TH levels in SN and ST of ACT001‐treated PD mice. (I) Brain G/M‐MDSCs of mice by the treatment of ACT001. (J) TGF‐β1 production of brain G‐MDSCs in ACT001‐treated PD mice (*n* = 6). All experiments were performed at least twice. Ns, no significance; *p < 0.05; **p < 0.01; ***p < 0.001.

