# Supplementary material for: GSDMD‐Deficient G‐MDSCs Exert Profoundly Suppressive Activity to Relieve MPTP‐Induced Parkinson's Disease
Source: CNS Neurosci Ther. 2025 Oct 13;31(10):e70626. doi: 10.1111/cns.70626 (PMC12518779; doi:10.1111/cns.70626)
Supplement: Supplementary file 1 — Figure S1: A representative FCM dot plot for MDSCs in the brain and spleen of PD mice. (A) Brain MDSCs were gated as CD11b + Gr‐1 + cells, G‐MDSCs gated as CD11b+ Ly6ClowLy6G+ cells, and M‐MDSCs gated as CD11b+ Ly6ChighLy6G−. (B) Spleen MDSCs and their typing. (C) Quantification of NLRP3, caspase‐1, cleaved caspase‐1, GSDMD, cleaved GSDMD and IL‐1β levels in G‐MDSCs from peripheral blood. Figure S2: Variations of TH levels in ST of MPTP‐treated mice. (A) Western‐blot analysis of α‐synuclein, TH, TNF‐α, and IL‐6 in striatum of WT or KO mice treated with MPTP. (B) Staining of TH in ST of WT or KO mice before and after the MPTP treatment. (C) Co‐staining of IBA‐1 with TNF‐α in ST of above mice. (D) Action trajectory of open field tests was documented among WT, GSDMD−/−, and Caspase 1−/− mice. (E) The TH variation of KO mice depleted with MDSCs by α‐DR5. Figure S3: Histological changes in ST of GSDMD‐Flox and GSDMD‐cKO mice. (A) Staining of ST. (B) Staining of TNF‐α and IL‐6. Figure S4: Histological changes in ST of MPTP‐treated mice infused with MDSCs. Staining of TH (A), IBA‐1 (B), and TNF‐α (C). Figure S5: Representative results of TNF‐α (A), IL‐6 (B), and IL‐1β (C) production by BV2 cells detected by flow cytometry. Figure S6: ACT001 promotes G‐MDSCs to inhibit PD progression. (A) Representative flow‐cytometric results of G/M‐MDSCs induced by ACT001 ex vivo. (B) Staining of TH in ST of PD mice treated with ACT001. [file CNS-31-e70626-s001.pdf]

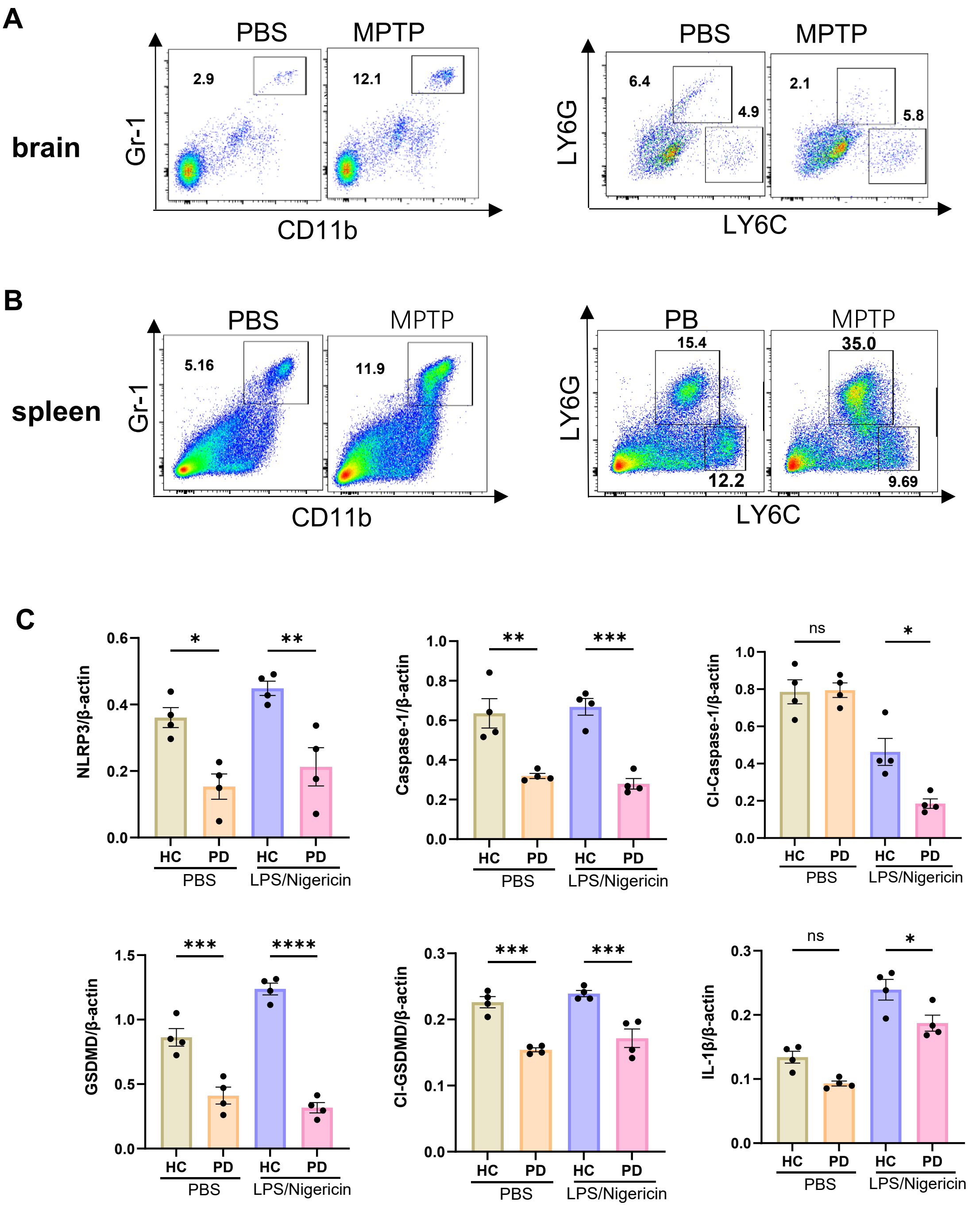

Fig. S1. A representative FCM dot plot for MDSCs in the brain and spleen of PD mice. **A.** Brain MDSCs were gated as CD11b$^+$Gr-1$^+$ cells, G-MDSCs gated as CD11b$^+$ Ly6C^{low}Ly6G$^+$ cells, and M-MDSCs gated as CD11b$^+$Ly6C^{high}Ly6G$^-$. **B.** Spleen MDSCs and their typing. **C.** Quantification of NLRP3, caspase-1, cleaved caspase-1, GSDMD, cleaved GSDMD and IL-1β levels in G-MDSCs from peripheral blood.

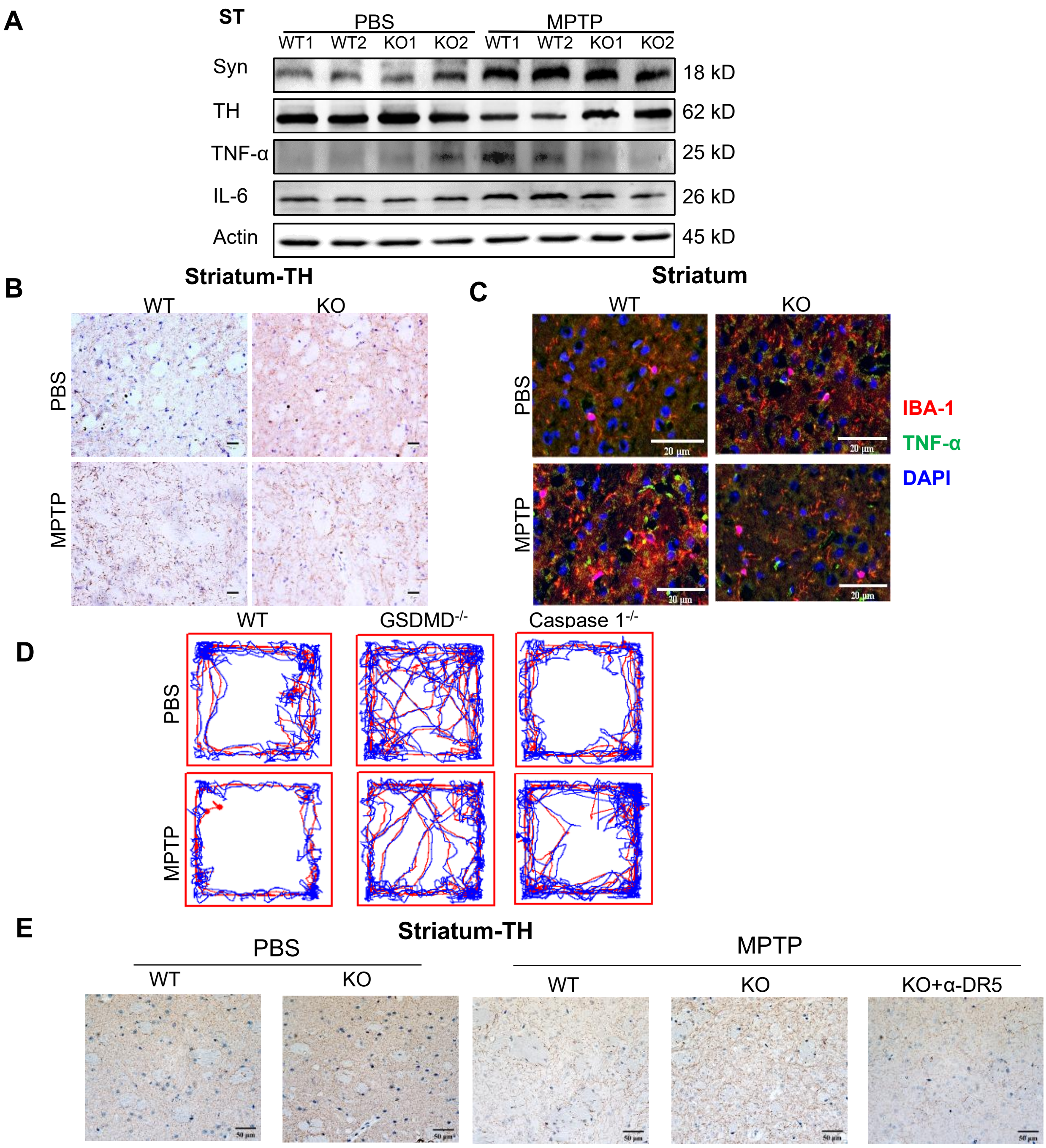

Fig. S2. Variations of TH levels in ST of MPTP-treated mice. **A**. Western-blot analysis of α-synuclein, TH, TNF-α, and IL-6 in striatum of WT or KO mice treated with MPTP. **B**. Staining of TH in ST of WT or KO mice before and after the MPTP treatment. **C**. Co-staining of IBA-1 with TNF-α in ST of above mice. **D**. Action trajectory of open field tests was documented among WT, GSDMD$^{-/-}$, and Caspase 1$^{-/-}$ mice. **E**. The TH variation of KO mice depleted with MDSCs by α-DR5.

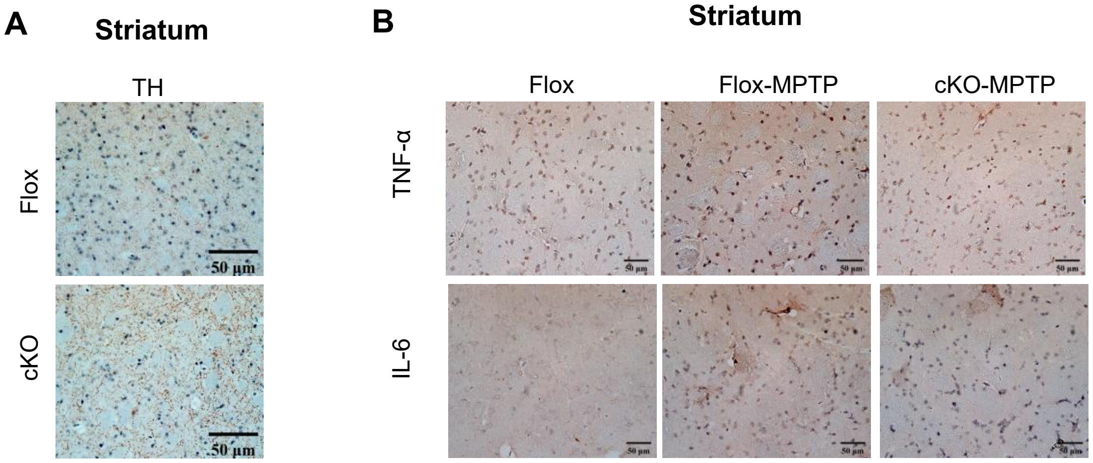

Fig. S3. Histological changes in ST of GSDMD-Flox and GSDMD-cKO mice. **A**. Staining of ST. **B**. Staining of TNF-α and IL-6.

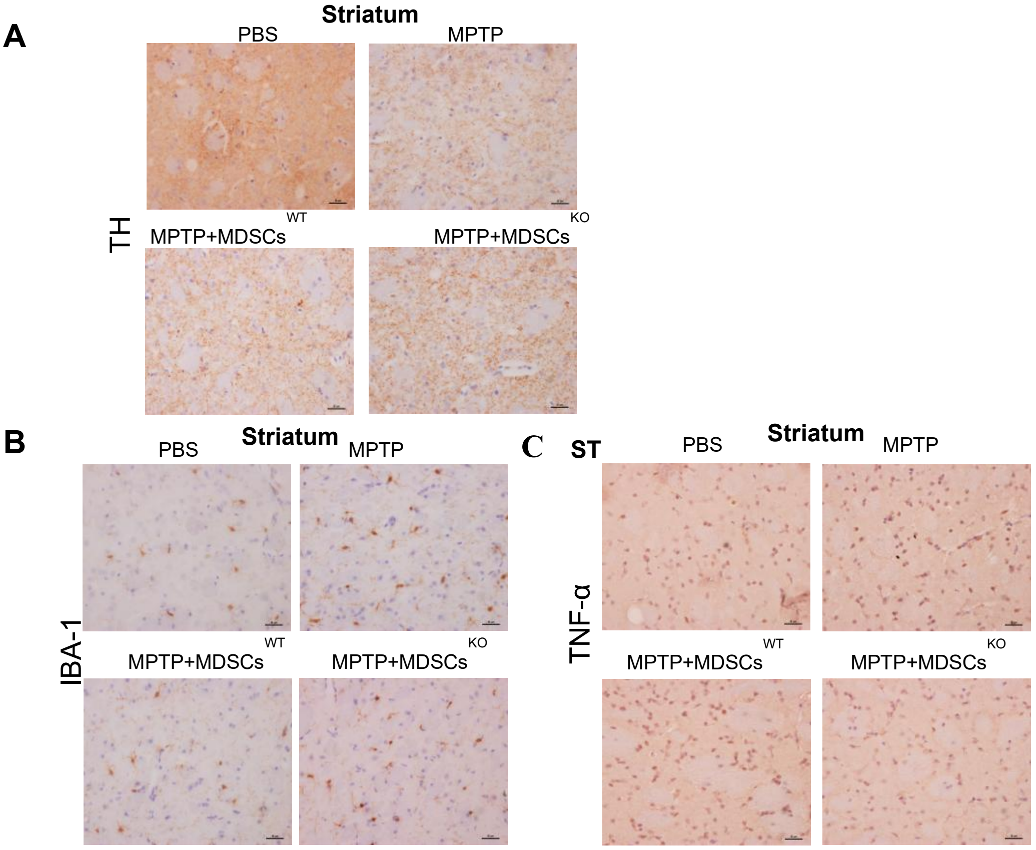

Fig. S4. Histological changes in ST of MPTP-treated mice infused with MDSCs. Staining of TH (**A**), IBA-1 (**B**), and TNF-α (**C**).

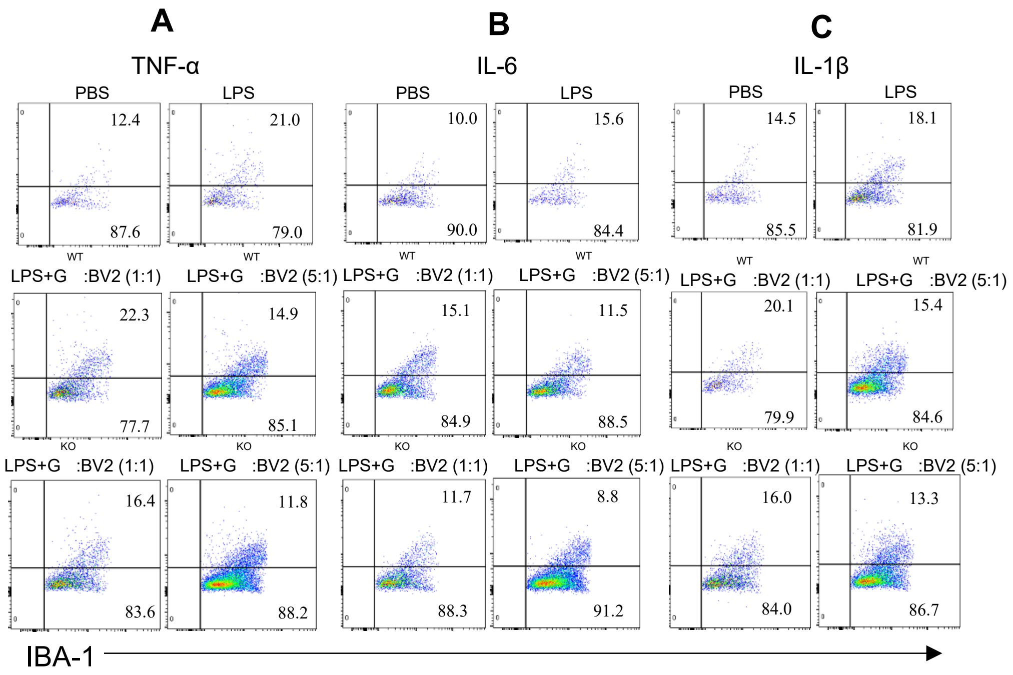

Fig. S5. Representative results of TNF-α (**A**), IL-6 (**B**), and IL-1β (**C**) production by BV2 cells detected by flow cytometry.

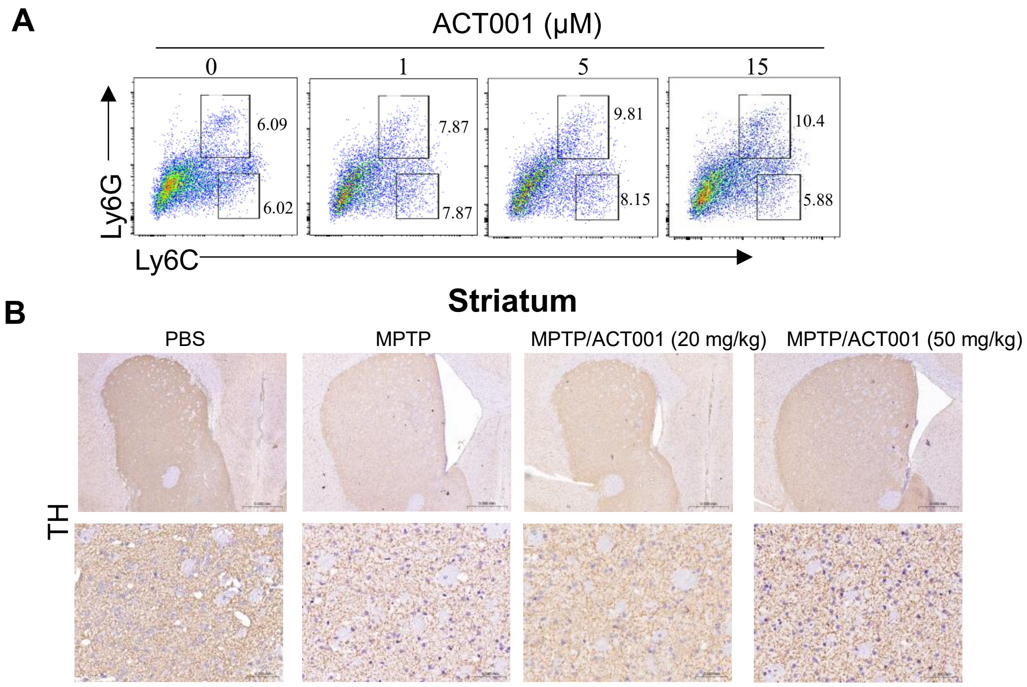

Fig. S6. ACT001 promotes G-MDSCs to inhibit PD progression. **A.** Representative flow-cytometric results of G/M-MDSCs induced by ACT001 ex vivo. **B**. Staining of TH in ST of PD mice treated with ACT001.
