# Supplementary material for: GSDMD‐Deficient G‐MDSCs Exert Profoundly Suppressive Activity to Relieve MPTP‐Induced Parkinson's Disease
Source: CNS Neurosci Ther. 2025 Oct 13;31(10):e70626. doi: 10.1111/cns.70626 (PMC12518779; doi:10.1111/cns.70626)
Supplement: Supplementary file 2 — Table S1: Clinical characteristics of PD patients and healthy controls (HC). [file CNS-31-e70626-s002.docx]

**Supplementary Table 1. Clinical characteristics of PD patients and healthy controls (HC)**

| **Variables** | **HC (n=21)** | **PD (n=37)** |
| --- | --- | --- |
| **Age** (years) | 65.62 ± 5.987 | 68.95 ± 7.059 |
| **Male** (%) | 11 (52.38 %) | 19 (51.35 %) |
| **Hypertension** (%) | 3 (14.28 %) | 14 (37.83 %) |
| **Diabetes** (%) | 2 (9.52 %) | 12 (32.43 %) |
| **Cerebral infarction** (%) | 0 | 6 (16.22 %) |
| **Hoehn-Yahr stage** | 0 | 2.5 (1.5-3) |
| **Educational attainment** | | |
| **Primary school** (%) | 7 (33.33 %) | 17 (45.95 %) |
| **Middle school** (%) | 11 (52.38 %) | 15 (40.54 %) |
| **High school** (%) | 3 (14.29 %) | 5 (13.51 %) |
| **Laboratory indexes at admission** | | |
| **Glucose**（mmol/l） | 5.14 ± 0.70 | 5.421 ± 2.13 |
| **HbA1c** (mmol/l) | 5.85 ± 0.49 | 6.313 ± 1.01 |
| **Homocysteine** (μmol/L) | 12.81 ± 6.75 | 11.24 ± 4.31 |
| **Uric acid** (μmol/L) | 276.0 ± 64.65 | 308.90 ± 90.34 |
| **ALT** (U/L) | 19.57 ± 5.65 | 17.51± 8.39 |
| **AST** (U/L) | 20.0 ± 4.20 | 21.68 ± 10.3 |
| **Serum creatinine** (μmol/L) | 68.74 ± 18.65 | 77.8 ± 20.75 |
| **BUN** (μmol/L) | 7.38 ± 3.36 | 6.43 ± 2.18 |
| **Triglyceride**（mmol/l） | 1.18 ± 0.34 | 1.35 ± 0.79 |
| **Total cholesterol**（mmol/l） | 4.03 ± 0.92 | 3.87 ± 1.08 |
| **HDL-c**（mmol/l） | 1.12 ± 0.30 | 1.22 ± 0.24 |
| **LDL-c**（mmol/l） | 2.46 ± 0.67 | 2.24 ± 0.99 |

Data are described as the mean ± SD, median (IQR), or n (%). ALT, alanine aminotransferase; AST, aspartate aminotransferase; BUN, blood urea nitrogen; HDL-c, high density lipoprotein cholesterol; LDL-c, low density lipoprotein cholesterol.
